# Transcatheter aortic valve implantation under lower activated clotting time in a patient with hemorrhagic gastric cancer: a case report

**DOI:** 10.1186/s40981-022-00566-9

**Published:** 2022-10-03

**Authors:** Yuki Mitsuta, Takafumi Oyoshi, Takahiro Nonaka, Naoyuki Hirata

**Affiliations:** 1 Department of Anesthesiology, Kumamoto University Hospital, Kumamoto, 860-8556 Japan; 2 Department of Anesthesiology, Kumamoto City Hospital, Kumamoto, Japan

**Keywords:** Transcatheter aortic valve implantation, Aortic stenosis, Activated clotting time

## Abstract

**Background:**

Perioperative management of transcatheter aortic valve implantation (TAVI) in patients with a high risk of bleeding requires careful consideration.

**Case presentation:**

A 74-year-old man complained of chest pain and was admitted to our hospital. Close examination revealed severe aortic stenosis (AS) and hemorrhagic gastric cancer. Hemorrhage from gastric cancer was controlled using endoscopic hemostasis. While both gastric cancer and AS required surgery, we decided to perform transfemoral transcatheter aortic valve implantation (TAVI) under monitored anesthesia. To reduce bleeding from gastric cancer, we carefully adjusted the heparin dose to maintain the activated clotting time (ACT) between 180 and 200 s. TAVI with a balloon-expandable valve was completed without thrombotic complications. Laparoscopic distal gastrectomy was performed on the 6th day after TAVI.

**Conclusions:**

We report the successful management of TAVI in a patient with hemorrhagic gastric cancer. In TAVI for patients with hemorrhagic diseases, careful consideration of antithrombotic therapy is required.

## Background

Symptomatic severe aortic stenosis (AS) is recommended for surgery unless it is clinically contraindicated or if the patient’s prognosis is less than 1 year [1]. The treatment strategy for patients with AS indicated for noncardiac surgery requires careful consideration. If patients with symptomatic severe AS have malignant tumors, the priority of the surgical procedure remains controversial. Postponing the surgery for a malignant tumor can worsen the malignancy, and perioperative antithrombotic therapy for surgical procedure for AS may result in critical hemorrhage from the tumor. Surgery for malignant tumors in patients with severe AS might induce critical cardiac events perioperatively. In this report, we describe a case in which transcatheter aortic valve implantation (TAVI) was performed on a patient with hemorrhagic gastric cancer.

## Case presentation

A 74-year-old man (height, 160 cm; weight, 55 kg) with a history of hypertension, dyslipidemia, and diabetes was admitted to our hospital complaining of chest pain. Transthoracic echocardiography (TTE) revealed severe AS [(aortic valve area: 0.5 cm^2^, maximum velocity (Vmax): 5.1 m/s, and mean pressure gradient (mPG): 67 mmHg]. Computed tomography revealed extravasation in the stomach. Upper gastrointestinal endoscopy revealed an ulcerative lesion in the lower body of the stomach and bleeding from the exposed blood vessels, and endoscopic hemostasis was performed. Based on the biopsy results, gastric adenocarcinoma was diagnosed, and surgery was indicated. We considered that the risks of cardiac events might be higher than the benefits of gastric surgery and decided to treat AS antecedently. We planned transfemoral TAVI under monitored anesthesia so the gastric cancer surgery could be performed as soon as possible after TAVI.

**Fig. 1:**
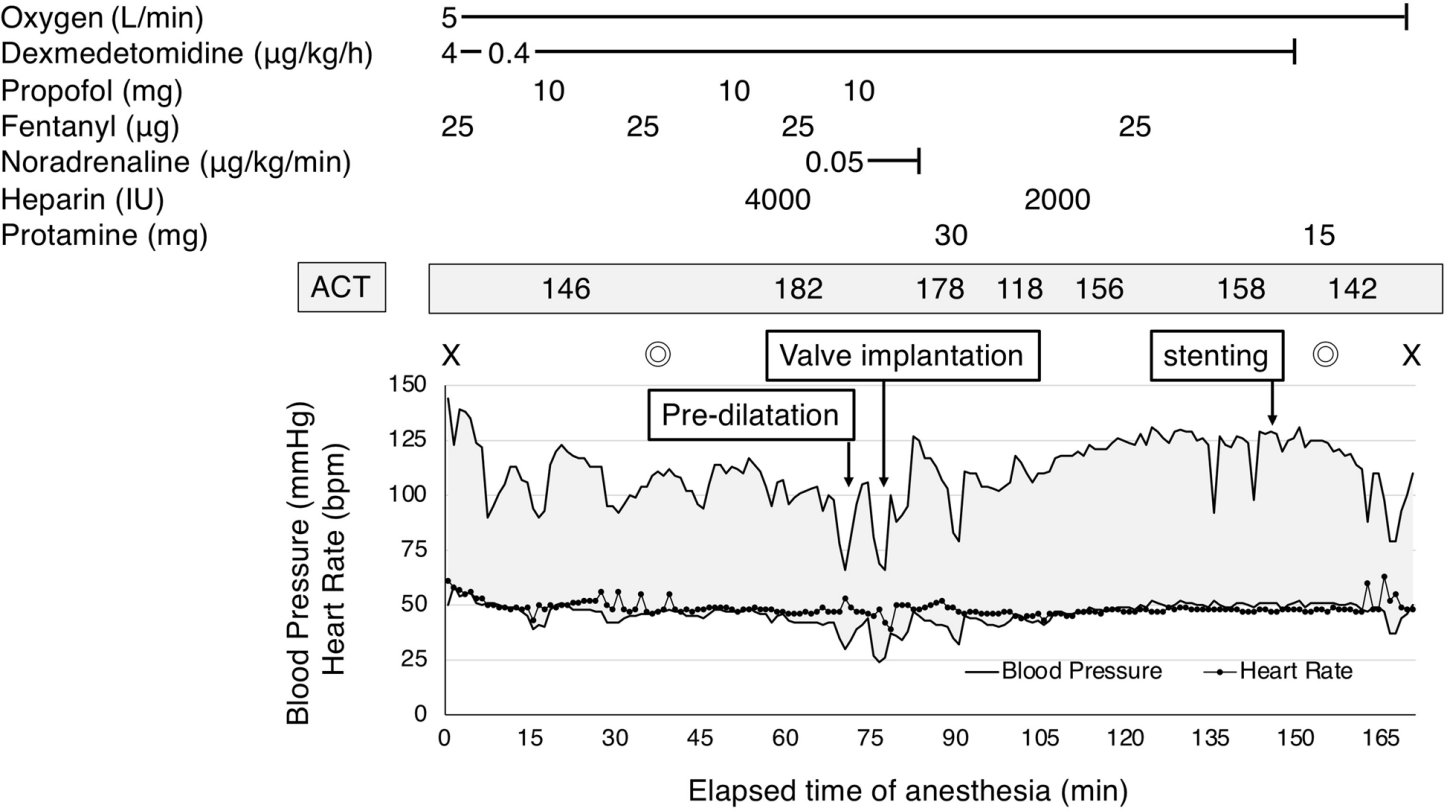
Anesthesia record. ACT, activated clotting time; X, start and end of anesthesia; ◎, start and end of operation

For monitored anesthesia, dexmedetomidine was loaded at 4 μg/kg/h for 10 min and maintained at 0.4 μg/kg/h. We used bispectral index (BIS; Bispectral Index™, Aspect Medical System Inc., Norwood, MA, USA) for evaluation of the level of anesthesia depth and near-infrared spectroscopy (INVOS 5100; Medtronic, Minneapolis, MN, USA) for evaluation of cerebral blood flow and oxygenation during the procedure. Propofol and fentanyl were administered as needed (Fig. 1), and lidocaine was injected at the puncture site.

We reduced the dose of heparin to 4000 units for controlling ACT between 180 and 200 s, shorter than 250 s commonly used during TAVI, based on 146 s before surgery in order to prevent exacerbation tumor bleeding, resulting in 182 s (Fig. 1). Pre-dilatation was performed under rapid pacing at 180 bpm, and a balloon-expandable valve (SAPIEN 3™) was implanted. Blood pressure gradually recovered, and TTE showed no pericardial fluid or paravalvular leak. Simultaneous pressure measurement showed an improved mPG of 18 mmHg. The re-examined ACT was 178 s, and protamine was administered, resulting in the ACT of 118 s. Perclose ProGlide™ devices were used at the access site but caused a narrowing of the right femoral artery. To improve femoral blood flow, a stent graft was placed using additional 2000 units of heparin. After the placement of stent graft with 156 s of ACT, the blood flow recovered. Administration of protamine resulted in ACT 142 s, and the surgery was completed. The patient recovered from sedation, and no neurological abnormalities were observed. The operation time was 124 min, the anesthesia time was 178 min, the bleeding volume was 50 ml, and 2 units of red blood cells were transfused during the operation.

Oral aspirin was administered on the day of the TAVI. TTE on the 5th day after TAVI showed that the prosthesis had no deposits and no deterioration of valve function: Vmax: 2.8 m/s and mPG: 16 mmHg. On the 6th day after TAVI, laparoscopic distal gastrectomy was performed under general anesthesia with a transfusion of 4 units of red blood cells. No postoperative events or complications occurred, and clopidogrel was started in addition to aspirin. The patient was discharged from our hospital on the 16th day after TAVI.

## Discussion

TAVI was performed before gastrectomy for patients with symptomatic severe AS and hemorrhagic gastric cancer.

The treatments for AS include balloon aortic valvuloplasty (BAV), surgical aortic valve replacement (SAVR), and TAVI. BAV has the advantage that the procedure is relatively simple and does not require postoperative antithrombotic therapy, but the procedure risk is not very low, and the improvement in severity may be insufficient [2]. SAVR is the gold standard for AS treatment in relatively young patients. However, considering the need for early gastric cancer surgery, TAVI is desirable because it has fewer postoperative complications in patients with malignancy [3], and the time to noncardiac surgery after TAVI may be shorter than that after SAVR [4]. Moreover, a high dose of heparin for extracorporeal circulation may exacerbate bleeding from gastric cancer. Taken together, we consider TAVI the best choice in this case.

In TAVI, heparin is administered, and ACT is generally maintained for 250 s or longer to prevent thrombus formation associated with intravascular procedures [5]. In this case, bleeding from gastric cancer continued, and it could be critical if the bleeding worsened due to heparinization. Therefore, the target ACT during TAVI was set to 180–200 s, which is lower than usual. There have been a report of shorter ACT (180–200 s) being applied for TAVI in a patient with pulmonary artery pseudoaneurysm [6] and a report of TAVI being performed with a shorter ACT (180–200 s) through carotid artery access under local anesthesia in 19 patients [7]. There have been two reports of TAVI being performed in patients with malignant tumors [8, 9]. In those reports, while the authors reported single antiplatelet therapy during malignant tumor surgery after TAVI, there was no description of ACT. Thus, there has been no report or evidence of TAVI being performed in a patient having a bleeding malignant tumor with a relatively short ACT as in our report. Further studies are needed to determine the safety margin of ACT for TAVI in a patients having the bleeding risk.

Current guidelines recommend dual antiplatelet therapy (DAPT) for 6 months, followed by single antiplatelet therapy (SAPT) for life [1]. In a study reported in 2020, the SAPT group had fewer bleeding events and a non-inferior composite of cardiovascular death, stroke, or myocardial infarction than the DAPT group [10]. In our case, SAPT was performed after TAVI, which led to gastric cancer surgery. No obvious exacerbation of tumor bleeding or thromboembolism was observed. SAPT should be performed in patients with high bleeding risk.

## Conclusions

In TAVI, for a patient with hemorrhagic gastric cancer, the intraoperative heparin dose was reduced, and ACT was kept lower to avoid exacerbation of tumor bleeding. Perioperative antithrombotic therapy for TAVI in patients at high risk of bleeding requires careful consideration.

## Data Availability

Data sharing is not applicable to this article as no datasets were generated or analyzed during the current study.

## Abbreviations

AS: Severe aortic stenosis
TAVI: Transcatheter aortic valve implantation
ACT: Activated clotting time
TTE: Transthoracic echocardiography
BAV: Balloon aortic valvuloplasty
SAVR: Surgical aortic valve replacement
DAPT: Dual antiplatelet therapy
SAPT: Single antiplatelet therapy
